# Prevalence and Hospitalization Trends of Cardiovascular Disease in Diabetic Populations: An Analysis of the United States Diabetes Surveillance System (USDSS) Data From 2000 to 2022

**DOI:** 10.7759/cureus.87886

**Published:** 2025-07-14

**Authors:** Efeturi M Okorigba, Omotola Akinade, Erhieyovbe Emore, Afolake A Adebayo, Oghenekome S Aghale, Edediong Ekarika, Kingdom Awuji, Oluseyi O Olawale, Theophilus Kutin Siaw, Regina U Azipu, Okelue E Okobi

**Affiliations:** 1 Internal Medicine, West Virginia University, Morgantown, USA; 2 Internal Medicine, General Hospital Ikorodu, Lagos, NGA; 3 Anatomy, Delta State University, Abraka, NGA; 4 Family Medicine, Nnamdi Azikiwe University, Nnewi, NGA; 5 Internal Medicine, Delta State University, Abraka, NGA; 6 Public Health, Emory University Rollins School of Public Health, Atlanta, USA; 7 Medicine, All Saints University School of Medicine, Roseau, DMA; 8 Internal Medicine, Ivano-Frankivsk National Medical University, Ivano-Frankivsk, UKR; 9 Psychiatry, Beaumont Psychiatric Clinic, Beaumont, USA; 10 Family and Community Medicine, Walden University, Minneapolis, USA; 11 Accident and Emergency, Korle Bu Teaching Hospital, Accra, GHA; 12 Internal Medicine, University of Calabar College of Medical Sciences, Calabar, NGA; 13 Family Medicine, Larkin Community Hospital Palm Springs Campus, Miami, USA

**Keywords:** cardiovascular disease, diabetes, disparities, epidemiology, hospitalization, prevalence, united states, usdss

## Abstract

Background: Cardiovascular disease (CVD) remains the leading cause of morbidity and mortality among individuals with diabetes. Understanding national time trends in both CVD prevalence and related hospitalizations is essential for informing clinical and public health strategies. This study aims to examine long‑term trends in CVD prevalence and hospitalization rates among US adults with diagnosed diabetes using the United States Diabetes Surveillance System (USDSS), which combines self‑reported survey data and administrative claims.

Methods: We conducted a retrospective observational analysis of USDSS data from 2000 to 2022. Adults aged ≥18 years with diagnosed diabetes were included. CVD prevalence was ascertained via self‑report or claims of coronary heart disease, myocardial infarction, or stroke. Annual crude hospitalization rates (per 1,000 diabetic individuals) were calculated using the total number of adults with diagnosed diabetes each year as the denominator; hospitalization data were available through 2020. Temporal trends were evaluated using linear trend analysis in addition to independent t‑tests (sex differences) and one‑way analysis of variance (ANOVA) (age and race/ethnicity disparities), with p < 0.05 denoting significance.

Results: From 2000 to 2022, major heart disease prevalence rose from 21.1% to 23.7% and then declined to 17.4%; however, absolute cases increased from 2.7 million to 4.9 million, a reflection of a growing diabetic population rather than a static denominator. Stroke prevalence peaked at 10.5% in 2008 before falling to 7.4% in 2022, while case counts doubled from 1.1 million to 2.1 million. Between 2000 and 2020, crude CVD hospitalization rates fell from 78.6 to 46.0 per 1,000 (data truncated at 2020 due to USDSS availability). Males consistently had higher rates than females (p < 0.05). Older adults (≥65 years) demonstrated the highest rates, with the ≥75 subgroup exhibiting the greatest absolute decline. Non‑Hispanic Black individuals experienced significantly higher hospitalization rates than other racial/ethnic groups (p < 0.05).

Conclusions: Although crude hospitalization rates for CVD among adults with diabetes declined over two decades, the absolute burden of disease rose in step with the expanding diabetic population. Significant disparities by age, sex, and race persist. Targeted interventions addressing both clinical care and population‑level drivers are needed for high‑risk diabetic subgroups.

## Introduction

Cardiovascular disease (CVD), encompassing coronary heart disease, myocardial infarction, and stroke, is the predominant cause of illness and death among individuals with diabetes mellitus (DM), representing a substantial public health concern both in the United States. The risk of coronary heart disease, stroke, and heart failure is significantly elevated in people with diabetes, particularly those with type 2 diabetes mellitus (T2DM) [1,2]. As of 2021, an estimated 537 million individuals worldwide are living with diabetes, and this figure is projected to increase to 783 million by 2045, positioning diabetes among the top ten causes of mortality in the United States. CVD remains the principal driver of complications and death in this population, with particularly high prevalence rates in regions such as North America and the Caribbean (46.0%) and Southeast Asia (42.5%) [3].

Adults diagnosed with diabetes are two to four times more likely to develop CVD than their nondiabetic counterparts [3]. This heightened risk results in more frequent hospitalizations, earlier mortality, and substantial increases in healthcare spending. In the United States, the Centers for Disease Control and Prevention (CDC) reported that, as of 2022, approximately 38.4 million Americans, equating to 11.6% of the population, had diabetes. T2DM accounts for the vast majority of these cases, exceeding 90% [4]. The prevalence rises sharply with age, affecting nearly 29.2% of adults aged 65 and older. Factors such as population aging, widespread obesity, physical inactivity, and socioenvironmental determinants are expected to further drive the rising incidence of diabetes.

Among individuals with diabetes, approximately one-third are estimated to have coexisting cardiovascular conditions, contributing to heightened clinical complexity and resource utilization [5]. According to the American Heart Association (AHA), nearly 70% of older adults (aged 65 and above) with diabetes had some form of heart disease in 2018, and 22% had experienced a stroke [6]. Hospital admissions for CVD in this population serve as both markers of disease severity and indicators of health‑system performance, though trends may also reflect evolving admission criteria or coding practices over time. These hospitalizations often reflect both acute events and the cumulative effects of long-standing metabolic dysfunction and inadequate disease management.

Although therapeutic advances, including cardioprotective glucose-lowering drugs, antihypertensive agents, lipid-lowering medications, and lifestyle-based interventions, have contributed to improved clinical outcomes, it remains uncertain whether these improvements have led to sustained reductions in CVD prevalence and hospital utilization across all demographic segments [7-9]. Previous research documented changes in diabetes‑related complications but did not leverage the United States Diabetes Surveillance System (USDSS) data to characterize long‑term national trends in both prevalence and hospitalization patterns across key demographic subgroups [10,11]. The USDSS uniquely integrates population‑level survey data, administrative claims, and hospital discharge records with consistent methodology from 2000 onward, making it ideally suited to track these trends.

This study therefore aims to (1) quantify temporal trends in CVD prevalence and crude hospitalization rates among US adults with diagnosed diabetes from 2000 through 2022 (hospitalization data through 2020); (2) evaluate differences by sex, age group, and race/ethnicity; and (3) discuss the implications of observed trends in light of both clinical advances (e.g., cardioprotective therapies) and potential administrative shifts (e.g., admission criteria, coding practices).

## Materials and methods

Study design and data source 

This retrospective observational study utilized publicly available data from the USDSS, a national database maintained by the CDC. The USDSS integrates multiple subdatasets, including prevalence data from the Behavioral Risk Factor Surveillance System (BRFSS) and the National Health Interview Survey (NHIS), both of which rely on self‑reported physician diagnoses, and hospitalization data from the National Inpatient Sample (NIS), an administrative‑claims database using the International Classification of Diseases, Ninth Revision (ICD‑9) and 10th Revision (ICD‑10) coding to capture inpatient discharges. Prevalence estimates extend through 2022, whereas hospitalization data end in 2020 because the NIS component of USDSS was only publicly released through calendar year 2020 at the time of analysis. All survey‑based prevalence inputs were weighted using CDC‑provided sampling weights to generate nationally representative estimates, and hospital discharge counts from NIS were similarly weighted to reflect national inpatient volumes.

Inclusion and exclusion criteria

Adults aged 18 years and older with diagnosed diabetes between 2000 and 2022 were identified by self‑report in BRFSS/NHIS, via responses to “Has a doctor ever told you that you have diabetes?” or by administrative claims in NIS using ICD‑9 codes 250.x and ICD‑10 codes E10-E14 in any diagnosis field. Individuals lacking a confirmed diabetes diagnosis, those with missing age, sex, race/ethnicity, or outcome variables, and those with institutionalized status were excluded. Hospitalization analyses were further restricted to discharges occurring between 2000 and 2020 with a primary CVD diagnosis defined by ICD‑9 codes 410-414 and 430-438 or ICD‑10 codes I20-I25 and I60-I69.

Study population and outcomes

The study cohort was stratified by sex (male and female), age group (18-44, 45-64, 65-74, and 75 years and older), and race/ethnicity (non‑Hispanic White, non‑Hispanic Black, Hispanic, and non‑Hispanic Asian/Pacific Islander). The first outcome, CVD prevalence, was defined as the percentage of adults with diabetes who reported or were coded with a history of coronary heart disease, myocardial infarction, or stroke. The second outcome, CVD hospitalization rate, was defined as the annual number of hospital discharges with a primary CVD diagnosis per 1,000 adults with diagnosed diabetes.

Statistical analysis

Survey‑derived prevalence estimates were calculated using CDC sampling weights to produce nationally representative metrics, while hospitalization rates were derived using NIS discharge weights. No additional age standardization was applied because age‑specific results are presented separately. Missing data, which accounted for less than 5% of records, were handled via complete‑case analysis, excluding individuals missing key covariates or outcomes. Temporal trends in prevalence and hospitalization rates were assessed using linear regression models with calendar year as the independent variable; model assumptions, including linearity, homoscedasticity, and normality of residuals, were evaluated through examination of residual plots and Shapiro-Wilk tests. Independent‑sample t‑tests compared mean hospitalization rates between males and females across the study period, and a one‑way analysis of variance (ANOVA) assessed differences across age and racial/ethnic subgroups. All statistical tests were two-sided, with p < 0.05 denoting significance. Data analysis was conducted in IBM SPSS Statistics for Windows, Version 30 (Released 2024; IBM Corp., Armonk, New York, United States), and visualizations were generated in MS Excel (Microsoft Corporation, Redmond, Washington, United States).

Ethical considerations

As this study was based on de-identified, publicly available data, it did not require institutional review board (IRB) approval under US federal regulations. All data were analyzed in accordance with the ethical standards of the CDC and the principles outlined in the Declaration of Helsinki.

## Results

Prevalence of CVD in diabetic populations

An analysis of USDSS prevalence data (BRFSS/NHIS) from 2000 to 2022 demonstrated a statistically significant downward linear trend in the prevalence of major heart disease among US adults with diagnosed diabetes (β = −0.27 percentage points per year; p = 3.7 × 10^-103). Model diagnostics indicated that assumptions of linearity, homoscedasticity, and normally distributed residuals were met. The prevalence rose modestly from 21.1% (95% CI: 18.9-23.5) in 2000 to 23.7% (95% CI: 21.4-26.1) in 2001, marking a brief inflection point, before declining steadily to 17.4% (95% CI: 15.7-19.3) by 2022. Although the proportion of affected individuals fell, absolute case counts increased from 2.7 million (95% CI: 2.4-3.0) in 2000 to 4.9 million (95% CI: 4.4-5.3) in 2022. This rise reflects the substantial growth of the diabetic population over the study period rather than an age‑standardized increase in risk; the lack of age standardization for absolute case numbers is acknowledged as a limitation.

Stroke prevalence exhibited greater year‑to‑year variability, peaking at 10.5% (95% CI: 9.0-12.2) in 2008 and declining to 7.4% (95% CI: 6.3-8.8) by 2022. A test for linear trend confirmed a small but significant downward slope (β = −0.10 pp per year; p = 5.4 × 10^-104^). Fluctuations around 2008 may reflect both changes in acute risk factors (e.g., atrial fibrillation management) and shifts in diagnostic coding practices. Despite proportional declines, the number of stroke cases doubled, from 1.1 million (95% CI: 0.9-1.3) in 2000 to 2.1 million (95% CI: 1.8-2.4) in 2022, again mirroring population growth rather than an age‑standardized incidence increase.

Overall, major heart disease prevalence declined at an average rate of 0.27 pp per year after 2001, and stroke prevalence declined by 0.10 pp per year after its 2008 peak. These inflection points highlight opportunities for targeted intervention early in the millennium. Table 1 summarizes these trends in both proportional and absolute terms.

**Table 1:** Major health disease and stroke prevalence over the years CI: confidence interval The values provided for prevalence in the table are averages with the range in parentheses

Year	Major heart disease		Stroke	
	Prevalence (%)	Total case in millions (95% CI)	Average prevalence (%)	Total case in millions (95% CI)
2000	21.1 (18.9-23.5)	2.7 (2.4-3.0)	8.7 (7.1-10.6)	1.1 (0.9-1.3)
2001	23.7 (21.4-26.1)	3.1 (2.8-3.5)	8.2 (7.0-9.5)	1.2 (1.0-1.3)
2002	23.1 (20.9-25.4)	3.3 (3.0-3.7)	8.9 (7.6-10.4)	1.3 (1.1-1.5)
2003	22.4 (20.4-24.6)	3.3 (3.0-3.7)	9.1 (7.8-10.7)	1.4 (1.2-1.7)
2004	21.5 (19.5-23.7)	3.5 (3.2-3.8)	8.2 (7.0-9.6)	1.4 (1.2-1.6)
2005	22.2 (20.2-24.5)	3.7 (3.3-4.1)	7.6 (6.4-8.9)	1.4 (1.1-1.6)
2006	21.8 (19.5-24.3)	3.9 (3.4-4.3)	7.2 (6.1-8.6)	1.5 (1.2-1.7)
2007	19.2 (17.1-21.5)	3.5 (3.1-4.0)	8.5 (7.1-10.2)	1.6 (1.3-1.8)
2008	22.0 (19.8-24.3)	4.3 (3.8-4.8)	10.5 (9.0-12.2)	2.1 (1.7-2.4)
2009	19.7 (17.7-21.8)	4.2 (3.7-4.6)	7.9 (6.7-9.3)	1.7 (1.4-2.0)
2010	21.5 (19.7-23.5)	4.7 (4.2-5.1)	9.1 (7.8-10.5)	1.9 (1.7-2.2)
2011	22.8 (21.0-24.7)	5.0 (4.6-5.4)	9.0 (7.8-10.4)	2.0 (1.7-2.2)
2012	21.4 (19.3-23.8)	4.7 (4.2-5.2)	8.9 (7.7–10.4)	2.0 (1.7–2.2)
2013	20.7 (19.0-22.5)	4.8 (4.4-5.2)	8.9 (7.6–10.4)	2.1 (1.8–2.4)
2014	18.2 (16.5-20.0)	4.3 (3.9-4.7)	8.2 (7.0-9.4)	1.9 (1.6-2.1)
2015	18.9 (17.2-20.8)	4.9 (4.4-5.4)	8.2 (7.1-9.5)	2.1 (1.8-2.4)
2016	18.4 (16.6-20.4)	4.5 (4.0-4.9)	7.9 (6.8-9.2)	2.0 (1.7-2.3)
2017	17.4 (15.6-19.5)	4.4 (3.9-4.8)	9.7 (8.1-11.5)	2.3 (1.9-2.6)
2018	18.3 (16.6-20.2)	5.2 (4.7-5.7)	8.7 (7.4-10.1)	2.4 (2.1-2.8)
2019	19.2 (17.5-21.0)	4.9 (4.5-5.4)	8.7 (7.5-10.1)	2.2 (1.9-2.4)
2020	17.1 (15.4-19.0)	4.7 (4.3-5.1)	6.7 (5.6-8.0)	1.8 (1.5-2.1)
2021	17.3 (15.8-19.0)	4.6 (4.2-5.1)	7.7 (6.6-9.1)	2.1 (1.8-2.3)
2022	17.4 (15.7-19.3)	4.9 (4.4-5.3)	7.4 (6.3-8.8)	2.1 (1.8-2.4)
p-value	p-value = 3.70E-103		p-value = 5.40E-104	
F value	F value = 1809224		F value = 1974762	

Trends in CVD hospitalization

Between 2000 and 2020, there was a substantial decline in the overall crude hospitalization rate for CVD among US adults with diabetes, decreasing from 78.6 per 1,000 in 2000 (95% CI: 72.9-84.3) to 46.0 per 1,000 in 2020 (95% CI: 43.0-49.0), reflecting an approximate 41% relative reduction. The most pronounced decrease occurred between 2000 and 2010, after which rates plateaued and fluctuated modestly within a 45-51 per 1,000 range. Notably, a temporary increase peaked at 51.9 per 1,000 in 2019, potentially driven by delayed treatment during that year’s healthcare access challenges and worsening cardiometabolic risk profiles in the prepandemic period, before falling again in 2020.

Correspondingly, the absolute number of CVD‑related hospital discharges rose from 1.54 million in 2000 to a peak of 1.92 million in 2019 and then declined to 1.68 million in 2020. This apparent paradox largely reflects a growing diabetic population, whose prevalence in the US increased from roughly 4.5% in 2000 to 11.6% by 2020, and an aging demographic, with the proportion of adults ≥65 years doubling during this period.

Cardioprotective therapies such as sodium-glucose cotransporter‑2 inhibitors (SGLT2is) and glucagon‑like peptide‑1 receptor agonists (GLP‑1 RAs) became widely adopted only after pivotal trials in the mid‑ to late‑2010s, limiting their impact on earlier hospitalization trends. While Empagliflozin Cardiovascular Outcome Event Trial in Type 2 Diabetes Mellitus Patients-Removing Excess Glucose (EMPA‑REG OUTCOME) (2015) and Liraglutide Effect and Action in Diabetes: Evaluation of Cardiovascular Outcome Results (LEADER) (2016) demonstrated reductions in heart failure hospitalizations, these agents comprised less than 5% of glucose‑lowering prescriptions by 2017. Their use remains disproportionately low among non‑Hispanic Black and Hispanic patients; fewer than 3% received SGLT2is in 2018 versus 7% of non‑Hispanic Whites, highlighting disparities in access and prescription.

Men consistently exhibited higher hospitalization rates than women (e.g., 81.7 vs. 75.4 per 1,000 in 2000), and the ≥75 years subgroup bore the highest absolute rates (peaking at 275.7 per 1,000 in 2001), underscoring the need for age‑focused preventive strategies. Non‑Hispanic Black adults experienced persistently higher rates than other groups, peaking at 82.4 per 1,000 in 2017, indicating a critical need for tailored interventions such as subsidized medication programs, deployment of mobile cardiometabolic clinics in underserved neighborhoods, and enforcement of prescribing guidelines that prioritize high‑risk minority populations. Table 2 below indicates the detailed annual rates and discharge counts.

**Table 2:** Trends in cardiovascular disease hospitalization during study period *: no data available; CI: confidence interval; suppressed: non-estimated value

Category	Year	2000	2001	2002	2003	2004	2005	2006	2007	2008	2009	2010	2011	2012	2013	2014	2015	2016	2017	2018	2019	2020	p-value	Statistical test value
Overall	Total-rate per 1000 (95% CI)	78.6 (72.9-84.3)	75.2 (69.8-80.5)	74.0 (68.2-79.8)	72.6 (67.1-78.1)	67.1 (62.3-71.8)	61.2 (56.6-65.8)	61.1 (55.9-66.2)	59.3 (54.1-64.5)	55.7 (50.8-60.6)	50.9 (46.9-54.9)	47.4 (43.9-50.8)	50.9 (47.3-54.5)	48.6 (46.0-51.3)	45.6 (43.3-48.0)	47.5 (44.9-50.2)	47.3 (44.6-50.1)	48.2 (44.9-51.5)	50.2 (46.9-53.5)	45.9 (43.4-48.5)	51.9 (48.7-55.0)	46.0 (43.0-49.0)	-	-
	Total-number of Discharges in 1000s (95% CI)	1535 (1463-1607)	1602 (1523-1681)	1612 (1525-1700)	1654 (1568-1739)	1617 (1537-1697)	1570 (1484-1656)	1671 (1573-1769)	1604 (1519-1689)	1620 (1527-1713)	1648 (1557-1740)	1569 (1490-1649)	1636 (1550-1722)	1618 (1577-1658)	1601 (1561-1641)	1614 (1575-1652)	Suppressed	1740 (1699-1780)	1825 (1783-1868)	1871 (1827-1915)	1920 (1874-1966)	1677 (1636-1717)	-	-
Based on gender	Male-rate per 1000 (95% CI)	81.7 (73.9-89.6)	79.3 (71.5-87.2)	77.1 (69.2-85.0)	78.1 (70.5-85.6)	73.2 (66.7-79.7)	68.5 (61.9-75.0)	69.3 (62.0-76.6)	67.1 (59.4-74.7)	63.8 (56.6-70.9)	54.3 (48.9-59.7)	50.5 (46.0-55.0)	55.9 (51.1-60.7)	55.5 (51.7-59.2)	51.7 (48.0-55.3)	53.2 (49.1-57.3)	55.4 (50.6-60.2)	55.0 (50.0-60.0)	53.5 (49.2-57.8)	51.4 (47.5-55.2)	59.8 (55.0-64.6)	51.7 (47.4-56.0)	p-value = 5.05E-12	T stat = -14.41
	Female-rate per 1000 (95% CI)	75.4 (68.7-82.0)	70.9 (65.1-76.8)	71.7 (65.2-78.2)	67.7 (61.7-73.7)	61.4 (56.2-66.7)	54.2 (49.6-58.9)	53.4 (48.3-58.4)	52.0 (46.9-57.0)	48.3 (43.5-53.1)	47.2 (43.0-51.4)	43.8 (39.9-47.6)	46.1 (42.2-49.9)	42.2 (39.1-45.2)	39.9 (37.3-42.5)	41.9 (39.0-44.8)	40.3 (37.5-43.1)	42.1 (38.6-45.6)	46.6 (42.8-50.4)	40.7 (37.7-43.6)	44.4 (41.0-47.9)	40.3 (36.7-43.8)		
Based on age	18-44 Rate per 1,000 (95% CI)	24.0 (20.4-27.6)	23.5 (20.4-26.6)	26.3 (22.4-30.1)	27.1 (23.0-31.1)	27.1 (23.4-30.8)	21.7 (18.5-24.8)	21.6 (18.3-25.0)	25.3 (20.8-29.8)	23.1 (18.8-27.3)	18.7 (15.8-21.5)	20.0 (17.1-22.8)	22.0 (19.0-25.0)	21.9 (19.0-24.8)	19.8 (17.3-22.3)	21.8 (18.8-24.7)	24.7 (21.2-28.2)	20.2 (16.8-23.5)	21.9 (18.7-25.2)	17.6 (15.1-20.1)	25.4 (21.8-28.9)	22.8 (19.2-26.5)	p-value = 1.28E-31	F value = 138.84
	45-64 Rate per 1,000 (95% CI)	97.0 (87.6-106.5)	86.0 (77.5-94.5)	86.3 (78.0-94.5)	87.6 (79.1-96.0)	77.2 (70.1-84.2)	68.4 (62.2-74.6)	72.9 (64.9-80.9)	66.8 (59.8-73.7)	58.1 (51.8-64.4)	55.3 (49.6-61.0)	54.5 (49.6-59.3)	55.6 (50.6-60.5)	51.7 (48.1-55.3)	51.6 (47.8-55.3)	52.9 (49.0-56.8)	51.5 (47.3-55.6)	56.1 (51.4-60.9)	55.2 (50.3-60.0)	57.5 (52.7-62.2)	60.1 (55.4-64.8)	57.6 (52.8-62.5)		
	65-74 Rate per 1,000 (95% CI)	167.4 (148.3-186.5)	163.9 (146.6-181.2)	156.4 (137.9-175.0)	149.8 (132.1-167.4)	136.2 (121.1-151.3)	128.1 (113.1-143.1)	133.5 (116.2-150.7)	115.6 (101.6-129.6)	114.2 (99.9-128.5)	112.2 (98.7-125.6)	94.1 (83.4-104.8)	93.3 (84.5-102.1)	94.7 (86.0-103.4)	87.4 (80.0-94.8)	82.6 (75.5-89.6)	81.0 (73.7-88.2)	81.7 (74.0-89.4)	96.3 (87.7-105.0)	84.8 (77.9-91.6)	94.3 (86.4-102.2)	75.5 (69.2-81.8)		
	75+ rate per 1,000 (95% CI)	269.1 (234.6-303.6)	275.7 (243.3-308.1)	250.3 (217.3-283.3)	230.5 (201.1-259.8)	215.0 (189.8-240.2)	219.4 (192.9-245.9)	195.5 (168.6-222.3)	192.0 (165.7-218.3)	196.1 (169.7-222.6)	177.8 (155.2-200.5)	148.8 (131.5-166.0)	176.0 (156.5-195.6)	161.7 (145.6-177.7)	146.4 (132.5-160.3)	157.5 (140.5-174.6)	142.8 (128.9-156.7)	165.7 (148.7-182.8)	166.0 (147.1-184.8)	145.9 (130.4-161.3)	148.0 (134.2-161.8)	122.2 (110.1-134.3)		
Based on race	Hispanic (95% CI)	*	*	*	*	*	*	*	*	*	*	*	*	33.4 (28.4-38.5)	31.8 (27.6-36.1)	32.2 (27.4-37.0)	31.6 (27.3-36.0)	32.8 (26.3-39.4)	32.3 (26.3-38.3)	34.3 (28.1-40.5)	36.5 (30.1-42.9)	29.5 (24.1-35.0)	p-value = 1.18E-19	F value = 167.88
	Non-Hispanic White (95% CI)	*	*	*	*	*	*	*	*	*	*	*	*	44.7 (41.5-47.9)	41.0 (38.2-43.8)	43.7 (40.6-46.8)	43.6 (40.1-47.2)	44.3 (40.7-47.8)	46.6 (43.1-50.1)	43.3 (40.2-46.4)	49.5 (45.7-53.3)	46.3 (42.3-50.3)		
	Non-Hispanic Black (95% CI)	*	*	*	*	*	*	*	*	*	*	*	*	63.5 (55.1-71.9)	63.9 (55.4-72.3)	64.2 (56.3-72.2)	66.9 (57.2-76.5)	66.8 (56.0-77.5)	82.4 (67.6-97.1)	72.0 (59.9-84.1)	78.7 (65.8-91.7)	63.9 (53.5-74.4)		
	Non-Hispanic Asian or PI (95% CI)	*	*	*	*	*	*	*	*	*	*	*	*	23.5 (19-28)	28.0 (21.5-34.4)	28.8 (22.1-35.5)	26.5 (20.3-32.6)	33.8 (24.4-43.2)	27.4 (20.7-34.0)	21.1 (16.7-25.5)	26.3 (20.2-32.3)	20.0 (15.7-24.3)		

Gender differences

**Figure 1:**
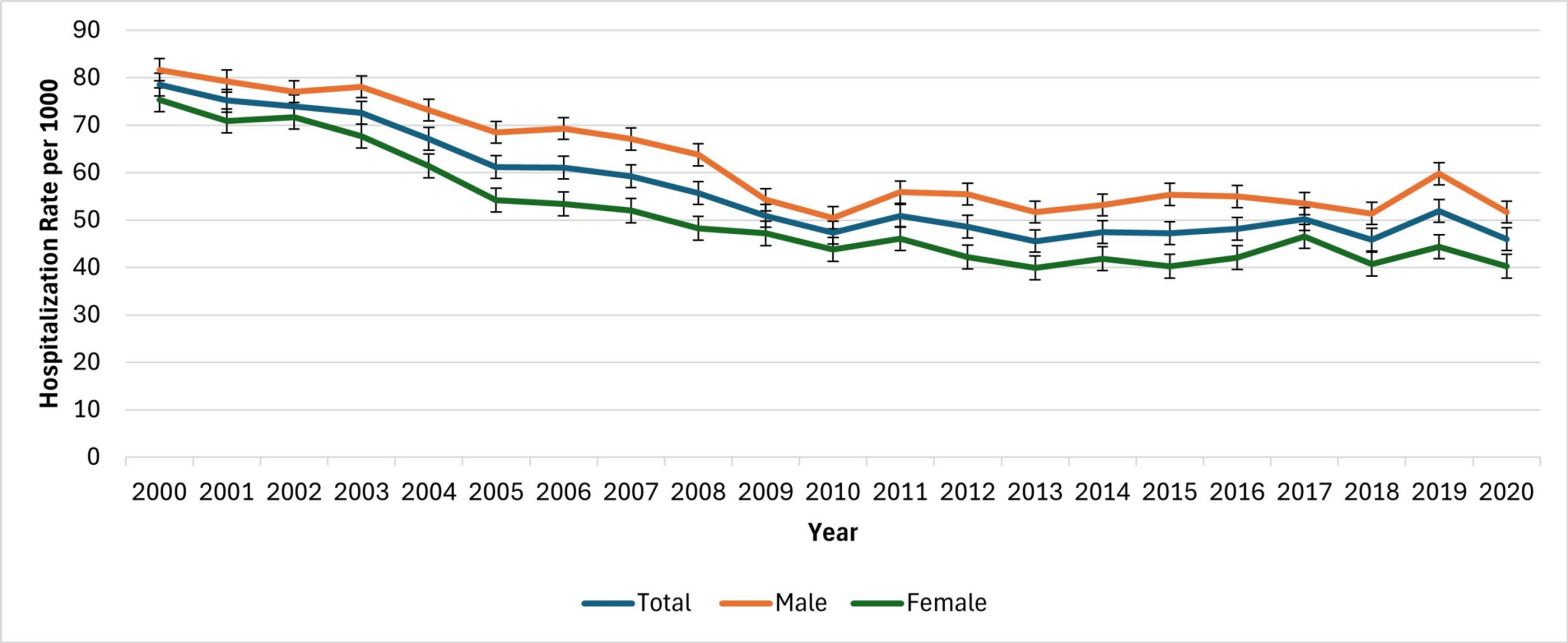
Cardiovascular disease hospitalization trends based on gender

When stratified by gender, men consistently exhibited higher crude hospitalization rates than women across 2000-2020 (Figure 1). In 2000, the rate among men was 81.7 per 1,000 (95% CI: 73.9-89.6), compared to 75.4 per 1,000 (95% CI: 68.7-82.0) among women. By 2020, rates had declined to 51.7 per 1,000 (95% CI: 47.4-56.0) for men and 40.3 per 1,000 (95% CI: 36.7-43.8) for women. The difference in trends between genders was statistically significant (independent sample t‑test, t = -14.41, p < 0.05), which may reflect differing risk profiles or patterns of healthcare utilization rather than a proven causal effect. Race/ethnicity analyses use a subset of NIS data reliably coded from 2012 onward; gender and age analyses span the full 2000-2020 period. Figure 1 below shows the CVD hospitalization trends based on gender. 

Age-specific trends

**Figure 2:**
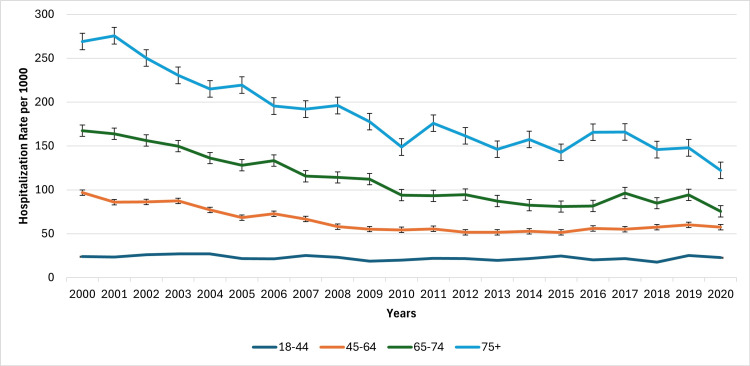
Cardiovascular disease hospitalization trends based on age

Hospitalization rates rose markedly with age and declined across all age categories over time (Figure 2). Adults aged 18-44 experienced the lowest rates throughout the period, dropping from 24.0 per 1,000 in 2000 to 22.8 in 2020 (p < 0.05; F = 138.84). Adults aged 45-64 saw a more substantial decline from 97.0 to 57.6 per 1,000, while those aged 65-74 decreased from 167.4 to 75.5 per 1,000. Among the oldest cohort (75+), hospitalization rates declined from a peak of 275.7 in 2001 to 122.2 in 2020. This group showed the largest absolute reduction, underscoring improved preventive care and possibly shifts in hospitalization criteria for older adults with diabetes. Figure 2 below indicates the CVD hospitalization trends based on age.

Racial and ethnic disparities

**Figure 3:**
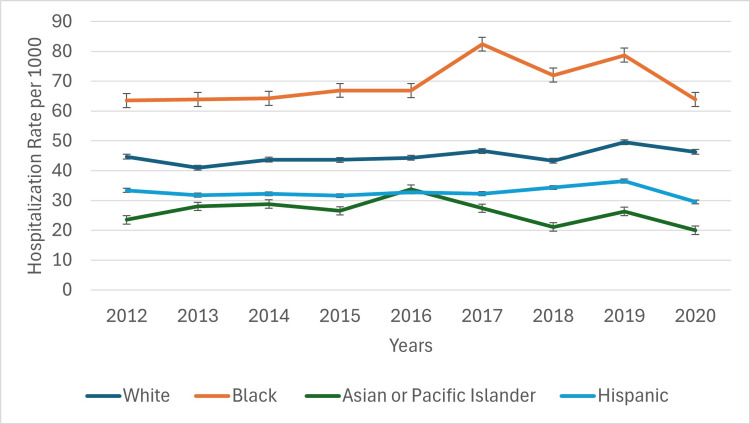
Cardiovascular disease hospitalization trends based on race

Between 2012 and 2020, CVD hospitalization rates among racial and ethnic groups exhibited persistent disparities (Figure 3). Non-Hispanic Black adults had the highest rates, increasing from 63.5 to a peak of 82.4 per 1,000 in 2017 before declining to 63.9 in 2020. In contrast, non-Hispanic White adults had moderately lower rates, ranging from 44.7 in 2012 to 46.3 in 2020. Hispanic adults consistently had lower hospitalization rates than other groups, declining slightly from 33.4 to 29.5 per 1,000 over the same period. Notably, non-Hispanic Asians and Pacific Islanders had the lowest rates, declining from 23.5 in 2012 to 20.0 in 2020. These differences were statistically significant (p < 0.05; F = 167.88), highlighting persistent inequities in CVD burden among adults with diabetes. Overall, these findings indicate progress in reducing CVD hospitalizations among diabetic adults but underscore continued disparities by age, gender, and race that warrant targeted public health interventions. Figure 3 below indicates CVD hospitalization trends based on race.

## Discussion

This study presents a detailed assessment of the CVD burden, specifically heart disease and stroke, among adults with diabetes in the US over two decades. The findings reveal complex trends in disease prevalence and hospitalizations, with progress in some areas yet a growing absolute burden and persistent disparities that continue to raise significant public health concerns.

While the age‑adjusted prevalence of heart disease and stroke among individuals with diabetes has declined, the absolute number of affected individuals has risen. This pattern, also reported by Gregg et al., reflects a growing diabetic population that could be the result of improvements in disease management [10]. Notably, the US population has not alleviated the rising absolute burden of cardiovascular complications among diabetic individuals, as noted in national surveillance data [11].

These proportional declines in CVD prevalence are likely attributable to several clinical advances. Improved glycemic control, stricter blood pressure and lipid management, and the widespread use of cardioprotective therapies have played key roles. Over the past two decades, clinical guidelines have increasingly recommended the use of statins, angiotensin-converting enzyme (ACE) inhibitors, or angiotensin receptor blockers (ARBs), and more recently, SGLT2is and GLP‑1 receptor agonists. Previous studies demonstrated that SGLT2 inhibitors provide consistent cardiovascular protection across various agents, highlighting their potential in diabetes care [12]. Nonetheless, the benefits of such interventions are being counteracted by population‑level drivers such as aging, rising obesity, and socioeconomic disparities that fuel diabetes incidence [12,13].

T2DM is prevalent and increasing among racial‑ethnic minority groups, especially the Black population. There are documented differences in medical care, such as fewer prescriptions for newer diabetes medications, poorer glycemic control, and reduced access to health resources due to insurance gaps, along with dietary patterns and higher hypertension rates. Lower educational attainment among some minority groups further compounds risk. Enhancing diabetes care in these communities could reduce complications and mortality from CVDs [14].

The impact of aging on diabetes includes increased comorbidity, polypharmacy, frailty, and limited access to preventive cardiology. Older adults with diabetes are at an elevated risk of CVD, including stroke, myocardial infarction, and heart failure [15]. Dyslipidemia, hypertension, and chronic kidney disease often accompany aging, necessitating multiple medications and increasing the risk of adverse events. Functional decline and social factors, such as transportation barriers and social isolation, can hinder timely care and contribute to hospitalizations [15].

Notably, stroke and heart disease demonstrated slightly divergent trajectories. Stroke prevalence exhibited greater fluctuations, potentially due to changes in acute risk factors or diagnostic coding [16,17]. However, the number of adults with diabetes affected by stroke nearly doubled during the study period, underscoring the need to prioritize cerebrovascular risk mitigation in diabetes care.

A key theme emerging from these findings is the association between improved clinical outcomes at the individual level and an increased disease burden at the population level. With the growing incidence of T2DM among younger adults, cumulative exposure to cardiovascular risk factors is lengthening [18].

Encouragingly, CVD‑related hospitalization rates declined from 2000 to 2020 among adults with diabetes, suggesting enhanced outpatient management and more effective use of preventive therapies. Landmark trials such as EMPA‑REG OUTCOME (2015) and DAPA‑HF (2019) demonstrated reductions in heart failure hospitalizations [19,20], and the LEADER trial (2016) showed decreased cardiovascular mortality with glucagon-like peptide-1 receptor agonists (GLP‑1 RAs) [21]. These timeline alignments suggest that the bulk of decline through 2010 reflects earlier interventions (statins, BP control), while mid‑2010s uptake of SGLT2is/GLP‑1 RAs may have contributed to continued, though more modest, gains. Our findings also align with Gregg et al., who documented decreases in major diabetes complications during a similar period [10]. Yet, absolute hospital discharges rose, reflecting the combined effects of rising diabetes prevalence and demographic shifts.

Men with diabetes experienced higher hospitalization rates than women, a trend established in epidemiological studies [22]. Biological factors, such as loss of estrogen‑mediated vascular protection after menopause, may partly explain this divergence, but evidence remains mixed and warrants cautious interpretation [23]. Differences in healthcare utilization may also play a role. Women with diabetes may face underdiagnosis or delayed referral due to atypical symptom presentation or implicit provider biases, as suggested by Kalyani et al. [23]. Insurance coverage gaps and lower referral rates for specialty care have been documented in some cohorts, potentially contributing to disparities in hospital presentation [23-26]. Behavioral factors, such as lower smoking cessation rates among men [27], and social determinants, including caregiving responsibilities, may influence healthcare‑seeking patterns. These remain hypotheses, as causal pathways are not fully established.

Besides, hospitalization rates declined across all age groups, with the largest absolute reductions among those ≥65 years. This likely reflects earlier and more intensive risk factor management in older adults through guideline‑driven screening and secondary prevention [15]. In contrast, limited improvement among young adults (18-44 years) mirrors rising obesity and youth‑onset diabetes, highlighting a need for early intervention [28]. Addressing persistent disparities requires precise interventions. For rural and low‑income minority communities, subsidized medication programs, measured by increases in SGLT2i/GLP‑1 RA prescription rates to >10% within two years, could improve uptake. Mobile cardiometabolic clinics offering screening and education in high‑burden ZIP codes, evaluated by annual reductions in hospitalization rates per 1,000 residents, would target underserved populations. Additionally, enforcing prescribing guidelines through electronic medical record alerts for patients meeting high‑risk criteria may reduce treatment gaps. Non‑Hispanic Black adults had the highest hospitalization rates, peaking at 82.4 per 1,000 in 2017. This two-to-threefold excess compared to Asian/Pacific Islander adults underscores the need for culturally tailored community interventions and policy reforms, such as Medicaid expansion in high‑disparity states, to improve access and outcomes [29,30].

In summary, while reductions in age‑adjusted prevalence and hospitalization rates reflect progress in individual‑level care, the growing absolute burden and persistent inequities signal ongoing systemic challenges. Future strategies must integrate clinical advances with public health interventions that address social determinants, healthcare delivery models, and provider practices to effectively reduce the cardiovascular toll of diabetes.

Strengths and limitations

A key strength of this study is its use of nationally representative data spanning two decades, allowing for a comprehensive evaluation of long‑term trends in CVD burden among adults with diabetes in the US. Prevalence estimates were derived from the BRFSS and NHIS, and hospitalization rates from the NIS (part of HCUP). The study’s retrospective observational design, leveraging existing USDSS sub-datasets, further ensures clarity in analyzing temporal trends. Stratified analyses by age, sex, and race/ethnicity increase awareness of disparities and inform targeted interventions. Additionally, integrating both prevalence and hospitalization data offers a nuanced view of clinical and public health dynamics, while the two‑decade span captures shifts in practice and policy.

However, the study has several limitations. First, hospitalization data may reflect changes in healthcare‑seeking behavior or access (e.g., shifts in admission thresholds or greater outpatient management) rather than underlying disease burden alone. Temporal changes in diagnostic practices, coding standards, or hospitalization criteria could likewise influence observed trends. Second, potential confounders, including population demographic shifts (e.g., aging, migration) and evolving comorbidity patterns, may impact longitudinal interpretations. Third, reliance on administrative and survey datasets may introduce coding inaccuracies, misclassification, and a lack of granular data on socioeconomic status, medication adherence, or access to care. Lastly, like many longitudinal studies, more recent trends may be underrepresented due to reporting lags or data completeness issues in the most recent USDSS releases. As with any observational analysis, causality cannot be established, warranting cautious interpretation of associations.

## Conclusions

This study highlights important trends in the cardiovascular burden among US adults with diabetes over the past two decades. While age-adjusted prevalence and hospitalization rates for heart disease and stroke have declined, the absolute number of affected individuals continues to rise, reflecting the expanding diabetic population. Disparities persist across sex, age, and racial/ethnic groups, underscoring gaps in equitable care delivery. These findings signal both progress in individual-level risk management and ongoing challenges at the population level.

Comprehensive strategies are essential to curb the rising absolute burden of CVD in people with diabetes. Clinically, this entails improved screening protocols (e.g., annual cardiovascular risk assessment beginning at diabetes diagnosis) and broader access to cardioprotective therapies, such as subsidizing SGLT2is or GLP‑1 RAs for underserved populations through insurance reforms or voucher programs. Early intervention should encompass both diabetes prevention (lifestyle modification programs for prediabetes) and early CVD detection (routine biomarker and imaging screening in high‑risk individuals). Public health actions might include mobile clinics delivering onsite screening in rural or minority communities and state‑level policies mandating coverage of intensive lifestyle and medication management under Medicaid.

Future policies must prioritize high‑risk groups, for example, by instituting sliding‑scale copayment waivers for cardioprotective drugs among low‑income adults or incentivizing primary care practices in Health Professional Shortage Areas to adopt guideline‑driven CVD screening metrics. Stakeholders can work toward meaningful, sustainable improvements in cardiovascular outcomes for people with diabetes by combining these targeted clinical and policy interventions with efforts to address social determinants of health (food insecurity, transportation barriers, and health literacy).
